# An observational study of a cohort of citizens receiving the AZD1222 vaccine against SARS-CoV-2

**DOI:** 10.2144/fsoa-2021-0064

**Published:** 2021-06-15

**Authors:** Pellegrino Cerino, Annachiara Coppola, Biancamaria Pierri, Palmiero Volzone, Dario Bruzzese, Andrea Pierri, Amedeo Ferro, Daniela Schiavi, Antonio Coppola, Stefano Miniero, Luigi Atripaldi, Caterina Pirozzi, Pasquale Rusciano, Alessandra Macri, Rita Boenzi, Silvia Sale, Gianfranco Brambilla, Carlo Buonerba

**Affiliations:** 1 Centro di Referenza Nazionale per l'Analisi e Studio di Correlazione tra Ambiente, Animale e Uomo, Istituto Zooprofilattico Sperimentale del Mezzogiorno, Portici, 80055, Italy; 2 Dipartimento di Medicina Sperimentale, Università degli studi della Campania ‘L Vanvitelli’, Naples, 80138, Italy; 3 Department of Medicine, Surgery & Dentistry (Scuola Medica Salernitana), University of Salerno, Baronissi, 84081, Italy; 4 Department of Public Health, University of Naples ‘Federico II’, Naples, 80131, Italy; 5 Cotugno Hospital, AORN Ospedali dei Colli, Naples, 80131, Italy; 6 ASL Napoli 3 SUD, Torre del Greco, 80059, Italy; 7 Food Safety, Nutrition & Veterinary Public Health Department, Istituto Superiore di Sanità, Rome, 00161, Italy

**Keywords:** adverse events, AZD1222, coagulation, COVID-19, SARS-CoV-2

## Abstract

In this retrospective study, a cohort of 67 subjects vaccinated with AZD1222 was retrospectively observed. Consistently with published findings, no serious adverse event was reported, and all adverse events reported (fever, muscle ache and/or pain in the site of injection) had resolved by day 8. Of note, some citizens were prescribed low-dose aspirin and even heparin for thrombosis prevention. We also found variations in laboratory test results (full blood count and chemistry) on day 1 compared with day 8. Physicians should be aware that no prevention therapy for thrombosis is currently recommended, given the very low incidence of this side effect. Additional studies are warranted to interpret our findings.

## Lay abstract

The AZD1222 vaccine against SARS-CoV-2 is effective in preventing COVID-19, but has been associated with rare, yet severe coagulation disorders. In this retrospective study, we reviewed available data obtained in a cohort of 67 citizens vaccinated with AZD1222. Four of them were prescribed low-dose aspirin or low-molecular-weight heparin for prevention of thrombosis by their family physician. Adverse events and biological parameters are also reported. Additional studies in larger cohorts are needed to interpret our findings.

As of 3 May 2021, SARS-CoV-2 has infected approximately 150 million individuals, and COVID-19 has caused about 3.2 million deaths worldwide [1]. While therapeutic options against COVID-19 remain limited, several well-conducted, randomized controlled population-based trials have led to the approval of a number of vaccines to prevent COVID-19, including mRNA vaccines (e.g., BNT162b2 [Pfizer–BioNTech], and mRNA-1273 SARS-CoV-2 [Moderna, MA, USA]) and viral vector vaccines (e.g., Sputnik V [Gamaleya Research Institute of Epidemiology and Microbiology, Moscow, Russia]; AZD1222 [AstraZeneca, Cambridge, England], developed by the University of Oxford; and Ad26.CoV2.S, [Johnson & Johnson, NJ, USA]) [2,3]. Although mass vaccination currently represents the backbone of the containment strategy for the SARS-CoV-2 pandemic, with over 1 billion vaccine doses administered, some serious adverse events – including fatal events – judged to be potentially associated with vaccine administration have been reported, especially for AZD1222. The AZD1222 vaccine is made of a replication-deficient chimpanzee adenoviral vector, ChAdOx1, incorporating the SARS-CoV-2 structural surface glycoprotein antigen gene [4]. The primary analysis of 17,178 citizens, of whom 8597 received AZD1222 and 8581 received the control intervention (meningococcal group A, C, W and Y conjugate vaccine or saline), showed an overall efficacy for AZD1222 of 66.7% in a large, randomized controlled clinical trial [5], with approximately 1% of individuals in both arms reporting a serious adverse event. After some sporadic cases of blood clotting disorders with low platelet count, including a few deaths, reported after administering AZD1222, several countries in the EU temporarily suspended its use, and others stopped the use of individual batches [6].

After reviewing 62 cases of cerebral venous sinus thrombosis and 24 cases of splanchnic vein thrombosis reported in the EU drug safety database as of 22 March 2021, 18 of which were fatal, the EMA’s safety committee concluded that a very low risk of blood clotting disorders with low platelet count does exist, although the benefits associated with AZD1222 largely overcome its risks [7]. Before the Italian Ministry of Health recommended that only citizens aged 60 or older should receive AZD1222, on 7 April 2021 [8], younger citizens, mostly teachers and police personnel, received AZD1222.

In this retrospective observational study involving citizens who received AZD1222 in March 2021, we reviewed available post-vaccination clinical and biological parameters in order to obtain potentially useful information in a common clinical practice scenario.

## Patients & methods

The purpose of this study was to explore patterns of drug use and adverse events associated with administration of the first dose of AZD1222. This retrospective observational study was conducted at the Local Health Unit of the southern area of the Metropolitan City of Naples (ASL Napoli 3 SUD). Citizens were included if data regarding drug use and adverse events reported after receiving the first dose of AZD1222 were available. The retrospective observation was conducted on days 1–8 after patients received the vaccine on day 0. Data regarding drug use and adverse events were retrieved along with demographic information and any biological parameters, if available, using an anonymized Excel datasheet. No ethics approval was obtained given the retrospective nature of this study.

Standard descriptive statistics were used to summarize biological parameters: mean ± standard deviation (min to max) or median (25th and 75th percentile; min to max) in case of variables showing substantial skewness. Accordingly, the significance of within-group changes was assessed using either the t test for paired samples or the Wilcoxon test and p-values <0.05 were considered statistically significant. No correction for multiple comparisons was applied. Statistical analyses were conducted using the R platform (v. 4.0.1; http://r-project.org).

## Results

A cohort of 67 adult citizens aged 24–65 years (median: 48; 56 females, 11 males) who received the first dose of AZD1222 in March 2021 were included in the study. All citizens were white, with the exception of one African–American woman, and received the same batch (ABV5811). Biological parameters and data regarding drug consumption and adverse events were collected on day 1 for the entire cohort and on day 8 for 57 patients. On day 1, the following adverse events were reported: shivers (14.9%), nausea (7.4%), muscle spasm (4.4%), body temperature >37.5°C (17.9%), fatigue (20.8%), headache (26.8%), pain in the site of injection (8.9%), muscle pain (23.8%) and abdominal muscle spasm (4.4%). Of note, data about post-vaccination drug consumption were available on day 1 for the entire cohort. A total of 28, four and two individuals reported consumption of paracetamol, aspirin and ibuprofen, respectively, to manage adverse events. Importantly, three patients were prescribed low-dose aspirin and one patient was prescribed low-molecular-weight heparin by their family physician for thrombosis prevention. All adverse events had resolved and drugs that had been started to manage symptoms or for thrombosis prevention on day 1 had been stopped by day 8. No serious adverse event was reported. Biological parameters assessed on days 1 and 8 were available for the entire cohort and for a subgroup of 57 individuals. All laboratory tests were performed at the Biochemistry Unit of the Monaldi Hospital. Biological parameters are reported in Table 1.

**Table 1.** Biological parameters.

	Day 1 (n = 67)	Day 8 (n = 57)	Change (95% CI); n = 57	p-value	Adjusted p-value	Increase or decrease
WBC (10^3^ μl)	5.98 ± 1.69 (2.64–10.76)	8.43 ± 1.97 (4.53–17.64)	2.54 (2.14–2.95)	<0.001	<0.001	↑
RBC (10^6^ μl)	4.72 [4.48; 4.93] (3.91–6.26)	4.81 [4.48; 5.04] (4.02–6.21)	0.05 (0–0.09)	0.031	0.437	
Lymphocytes (10^3^/μl)	1.39 [1.1; 1.78] (0.55–3.05)	2.57 [2.26; 3.13] (1.49–6.49)	1.23 (1.08–1.41)	<0.001	<0.001	↑
Monocytes (10^3^/μl)	0.71 ± 0.22 (0.34–1.37)	0.6 ± 0.16 (0.32–1.09)	-0.12 (-0.16 to -0.08)	<0.001	<0.001	↓
Neutrophils (10^3^/μl)	3.7 ± 1.37 (1.26–7.03)	4.86 ± 1.31 (1.7–9.67)	1.26 (1–1.52)	<0.001	<0.001	↑
Eosinophils (10^3^/μl)	6 [2; 10.5] (0–36)	12 [8; 22] (0–49)	8 (6–10.5)	<0.001	<0.001	↑
Basophils (10^3^/μl)	0.04 ± 0.02 (0.01–0.12)	0.05 ± 0.02 (0.02–0.12)	0.01 (0.006–0.013)	<0.001	<0.001	↑
PLT (10^3^/μl)	237.7 ± 49.3 (140–344)	299.4 ± 67.8 (179–484)	63.8 (51.5–76.1)	<0.001	<0.001	↑
HGB (g/dl)	13.7 ± 1.3 (7.9–18.1)	13.9 ± 1.5 (7.8–18)	0.1 (-0.2 to 0.4)	0.427	1	
APTT (s)	30.6 [28.6; 32.2] (25.8–56.1)	30.8 [29.4; 32.7] (26.1–56.5)	0.5 (-0.1 to 1.1)	0.148	1	
PT (s)	11.5 ± 0.7 (10.1–13.6)	10.7 ± 0.6 (9.6–12)	-0.8 (-1 to -0.6)	<0.001	<0.001	↓
Uremia	29.7 ± 7 (17–47)	33.4 ± 7.2 (19–49)	4 (2.5–6)	<0.001	<0.001	↑
Total bilirubin (mg/dl)	0.51 [0.4; 0.82] (0.17–2.01)	0.52 [0.4; 0.8] (0.17–2.06)	-0.02 (-0.06 to 0.02)	0.332	1	
Calcium (mg/dl)	10.1 ± 0.5 (9–11)	10.5 ± 0.3 (9.7–11.6)	0.4 (0.3–0.6)	<0.001	<0.001	↑
Creatinine (mg/dl)	0.7 [0.6; 0.7] (0.4–1.3)	0.6 [0.5; 0.7] (0.5–1)	0 (-0.1 to 0)	0.15	1	
D-dimer (ng/dl)	115 [84; 163.5] (44–460)	97 [76; 132] (30–351)	-27 (-38.5 to -15)	<0.001	0.001	↓
Fibrinogen (mg/dl)	323.2 ± 61.9 (231–527)	289.4 ± 55.6 (207–459)	-32.2 (-44.5 to -19.8)	<0.001	<0.001	↓
Alkaline phosphatase (U/l)	71.5 ± 18.3 (40–121)	72.5 ± 16.4 (38–107)	0.6 (-1.2 to 2.4)	0.483	1	
GGT (U/l)	17 [12; 26] (7–119)	18 [14; 27] (7–104)	1.5 (0.5–2.5)	0.001	0.028	↑
AST (U/l)	21 [19; 25] (14–66)	22 [19; 26] (14–121)	1 (-0.5 to 2.5)	0.154	1	
ALT (U/l)	19 [15; 33] (8–96)	20 [16; 30] (10–221)	0.5 (-1 to 2)	0.653	1	
LDH (g/dl)	187.2 ± 28.8 (136–279)	192.7 ± 28.7 (149–289)	8.2 (3–13.4)	0.003	0.043	↑
Potassium (mEq/l)	4.4 [4.2; 4.7] (3.4–6.4)	4.4 [4.3; 4.7] (3.5–6.4)	0 (-0.1 to 0.2)	0.73	1	
Sodium (mEq/l)	140 [139; 141] (137–144)	140 [138; 141] (130–144)	-0.5 (-1.5 to 0)	0.08	1	

Data are expressed as mean ± standard deviation (min to max) or as median [25th; 75th percentile] (min to max).

ALT: Alanine aminotransferase; APTT: Activated partial thromboplastin time; AST: Aspartate aminotransferase; GGT: γ-glutamyl transferase; HGB: Hemoglobin; LDH: Lactate dehydrogenase; PLT: Platelets; PT: Prothrombin time; RBC: Red blood cell; WBC: White blood cell.

## Discussion

In the analysis of safety data on 74,341 person-months of follow-up after first dose and 29,060 person-months of follow-up after two doses, serious adverse events were reported in 168 individuals, 79 in the AZD1222 arm and 89 in the control arm [4]. Only three events were considered possibly related to either the experimental or the control intervention and included a case of hemolytic anemia in the control group, a case of transverse myelitis after AZD1222 booster vaccination and a case of fever higher than 40°C in a participant who was masked to group allocation at the time of the report [4]. Of note, there was no excess of thrombotic disorders associated with AZD1222 in the published clinical trials. A review of available population-based data did conclude that AZD1222 administration could be rarely associated with onset of a potentially fatal adverse event named ‘thrombosis with thrombocytopenia syndrome’ (TTS), which presents itself with blood clotting disorders associated with low platelet counts [9]. Although the estimated incidence is 1 case per 250,000 vaccinated individuals [9], some countries (e.g., Denmark) have ceased AZD1222 use completely [10], while others (e.g., Germany) have temporarily restricted its use to citizens >60 years old [11], as TTS seems to be associated with younger age.

In our small retrospective study, we aimed to gather all available data obtained in adult citizens who received the first dose of AZD1222 while fears of potential serious blood clotting disorders induced by the vaccine were growing in the public opinion. One important finding is that we gathered evidence of use of low-dose acetylsalicylic acid and even heparin that were specifically prescribed for thrombosis prevention, which the National Drug Agency has regarded as inappropriate [12] (independent of possible risk factors that may have been considered on an individual basis by the prescribing physicians) given the very low incidence of TTS and the absence of established risk factors.

Laboratory tests performed on days 1 and 8 showed mean variations of several parameters. The statistically significant differences in laboratory tests found may simply be the result of chance or of the immune response elicited by the vaccine, although additional studies in larger cohorts are needed to interpret our findings. The main limitations of our study include the lack of a baseline assessment (right before vaccination) and the small sample size, which does not allow us to extrapolate our results to the general population without confirmation in larger studies.

## Conclusion

Physicians should be warned that there is currently no indication for preventive use of anticoagulant agents in subjects undergoing vaccination with AZD1222 or any other anti-SARS-CoV-2 vaccine. Off-label use of anticoagulant agents in vaccinated subjects should be monitored (e.g., by questioning patients at the time of the second vaccine administration).

## Future perspective

In the current scenario, it can be expected that vaccines based on viral vectors will be less and less extensively used for mass vaccination campaigns, which will be primarily conducted using mRNA-based vaccines. Major objectives include the need to reduce the cost of mRNA-based vaccines as well as to monitor the effectiveness of such vaccines toward novel SARS-CoV-2 variants.

**Executive summary**

- The purpose of this study was to explore patterns of drug use and adverse events associated with administration of the first dose of AZD1222.
- A cohort of 67 adult citizens aged 24–65 years (median: 48; 56 females, 11 males) who received the first dose of AZD1222 in March 2021 were included in the study.
- A total of 28, four and two individuals reported consumption of paracetamol, aspirin and ibuprofen, respectively, to manage adverse events, while three patients were prescribed low-dose aspirin and one patient was prescribed low-molecular-weight heparin by their family physician for thrombosis prevention.
- No serious adverse event was reported, while we found variation in most biological parameters of the blood on day 8 compared with day 1 after vaccination.
- Additional studies are required to interpret our findings.
